# Organization of human replicon: Singles or zipping couples?

**DOI:** 10.1016/j.jsb.2008.11.004

**Published:** 2009-03

**Authors:** Anna Ligasová, Ivan Raška, Karel Koberna

**Affiliations:** a Laboratory of Cell Biology, Institute of Experimental Medicine, v.v.i., Academy of Sciences of the Czech Republic, Vídeňská 1083, 14200 Prague 4, Czech Republic; b Institute of Cellular Biology and Pathology, First Faculty of Medicine, Charles University in Prague and Department of Cell Biology, Institute of Physiology, Academy of Sciences of the Czech Republic, v.v.i., Albertov 4, 12800 Prague, Czech Republic

**Keywords:** Chromatin, Replication, Replicons, Replisomes

## Abstract

According to a general paradigm, proper DNA duplication from each replication origin is ensured by two protein complexes termed replisomes. In prokaryotes and in budding yeast *Saccharomyces cerevisiae*, these two replisomes seem to be associated with one another until DNA replication initiated from the origin has finished. This arrangement results in the formation of the loop of newly synthesized DNA. However, arrangement of replisomes in other eukaryotic organisms including vertebrate cells is largely unknown. Here, we used in vivo labeling of DNA segments in combination with the electron microscopy tomography to describe the organization of replisomes in human HeLa cells. The experiments were devised in order to distinguish between a model of independent replisomes and a model of replisome couples. The comparative analysis of short segments of replicons labeled in pulse-chase experiments of various length shows that replisomes in HeLa cells are organized into the couples during DNA replication. Moreover, our data enabled to suggest a new model of the organization of replicated DNA. According to this model, replisome couples produce loop with the associated arms in the form of four tightly associated 30 nm fibers.

## 1 Introduction

During the replication of both prokaryotic and eukaryotic genomes, two replication complexes, commonly called replisomes, are believed to be established at the origins of the replication. According to this widely accepted scheme, the synthesis of DNA in opposite directions from the replication origin is ensured by couples of “sister” replisomes (Baker and Bell, 1998; Waga and Stillman, 1998; Johnson and O’Donnell, 2005). Two basic arrangements of a replisome couple during the DNA replication have been suggested earlier. In the first one, “sister” replisomes move along the DNA molecule in opposite directions. The second one is based on a tight association of replisomes in a replisome couple. This organization results into a transient formation of a loop consisting of newly synthesized DNA. Although the model of tightly associated “sister” replisomes was suggested already in 1974 (Dingman, 1974), the first convincing proof of such arrangement was not available until much later. The fact that prokaryotic cells use this type of replisome organization has been indicated by several recent findings (Lemon and Grossman, 2000; Jensen et al., 2001; Lau et al., 2003; Migocki et al., 2004). However, the only supporting data for such organization in eukaryotic cells has been yet provided in budding yeast *Saccharomyces cerevisiae* (Kitamura et al., 2006, commented by Meister et al., 2006).

The DNA of vertebrates is replicated via a large number of DNA segments termed as replicons, which are continuously activated in the S phase (Edenberg and Huberman, 1975; Hand, 1978). The size of the individual replicons usually varies from 30 to 450 kbp, with the most frequent size being 75–175 kbp, although replicons below 10 kbp and above 1 Mbp have also been described (Edenberg and Huberman, 1975; Yurov and Liapunova, 1977; Hand, 1978; Hyrien and Mechali, 1993; Jackson and Pombo, 1998; Berezney et al., 2000). Based on studies of stretched DNA fibers, it is supposed that clusters of adjacent replicons are usually synchronously activated and jointly ensure the replication of several hundreds of kilobases of DNA (Edenberg and Huberman, 1975; Hand, 1978). The number of replicons in one such replicon cluster varies but is usually less than 10 (Jackson and Pombo, 1998; Ma et al., 1998). In situ, replicon clusters are commonly identified with replication foci (light microscopy/LM entities) or replication factories (electron microscopy/EM entities), structures which can be observed after immunocytochemical detection of DNA synthetic activity (Nakamura et al., 1986; Nakayasu and Berezney, 1989; Fox et al., 1991; O’Keefe et al., 1992; Hozak et al., 1993; Ma et al., 1998; Dimitrova and Gilbert, 1999; Leonhardt et al., 2000).

**Fig. 1:**
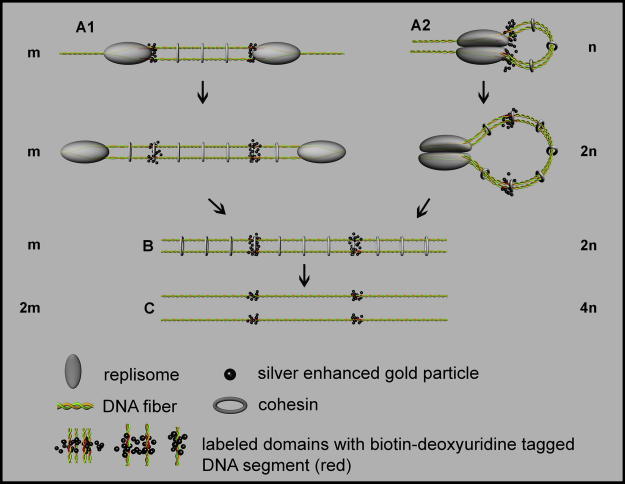
The explanatory scheme depicting two models of the arrangement of “sister” replisomes in HeLa cells and the effect of different organizations of biotin-dU-tagged segments on the number of labeled domains during various pulse-chase experiments. The scheme shows the expected results of the consecutive mapping (indicated by arrows) of segments tagged during a short pulse of biotin-dUTP in early S phase followed by the chase period of different length from the time immediately after the pulse (the upper part of the scheme) to the complete mitotic segregation of the sister chromatids (the lower part of the scheme). Note that some clusters of silver-enhanced gold particles belonging to the mutually close segments can “fuse”. Therefore, the domain labeled by silver-enhanced gold particles, used as markers in the present study, can contain between one and four segments, depending on the model and the length of the chase. This “fusion” is a result of the “large” size of the antibody complex with the silver-enhanced gold particle as against the distance between segments. The expected number of domains for the individual stages of replicon organization is shown as a multiple of initial number of domains. The initial number is designated by m for the model of replisome singles and by n for the model of replisome couples. Note that the number of labeled domains is doubled in the model of replisome singles (A1) and quadrupled in the model of replisome couples (A2) in mitosis. In fact, the increase in the model of replisome couples is lower as labeled segments of replicons early after initiation cannot contribute to this increase (see below). Several simplifications have been used in the model such as chromatin is shown as a DNA double helix in all the models although the DNA in chromatin is more condensed. In addition, the partial segregation of chromatids is not taken into account in the model before mitosis. (A1) A model of replisome singles. “Sister” replisomes move in opposite directions during replication. The two tagged segments of the sister chromatids are close to each other both during and after replication due to the cohesion of the sister chromatids mediated by a cohesin complex. Each labeled domain contains one pair of “sister” segments. The number of the labeled domains remains unaltered during this process. (A2) A model of replisome couples. “Sister” replisomes form a closely associated complex, resulting in the formation of a DNA loop. The four tagged segments are in close proximity at the time of their replication and are visualized as one labeled domain. Later, the loop inflates and consequently, the distance between both “sister” pairs of the tagged segments of chromatids is gradually prolonged and the number of labeled domains increases. Each labeled domain contains only one pair of segments at this point. (B) Two sister chromatids bound together by cohesin complexes after the termination of replicon synthesis and dissociation of replisomes are shown. No difference in the organization of the tagged segments is visible in the case of the model of replisome singles. The number of the labeled domains is also the same when compared with the ongoing replicon replication shown in A1. On the other hand, the relaxation of the loops shown in the model of replisome couples (A2) resulted in an increase in distances between the pairs of tagged chromatin segments, which facilitates the recognition of previously less distant “sister” pairs. Consequently, the number of the labeled domains is nearly doubled with respect to the number of domains found immediately after the biotin-dUTP labeling pulse. The increase is lower as labeled segments of replicons which began DNA synthesis during the pulse are not separated by non-labeled DNA strand. (C) In mitosis, sister chromatid cohesion is broken and the pairs of the tagged segments separate. Mitotic segregation results in the twofold increase of labeled domains with respect to (B). Each individual domain contains only one biotin-dU-tagged chromatin segment.

In this study, we have designed experiments capable of distinguishing between the two models of replisome arrangement based on the pulse-chase experiments of various lengths (see Fig. 1). We have visualized short segments of active replicons by replication-mediated labeling with biotin-16-2′-deoxy-uridine-5′-triphosphate (biotin-dUTP) followed by immunocytochemical detection of incorporated biotin-16-2′-deoxy-uridine (biotin-dU). Biotin-dUTP was selected from various nucleotide analogues as this nucleotide analogue does not require a cell-structure damaging steps such as treatment with concentrated acid. This rough treatment is necessary for the visualization of halogen derivatives of nucleosides that are commonly used in LM experiments. We have used the pre-embedding labeling for the localization of biotin-dU inside the sections as it allowed us to analyze the signal by means of EM tomography approach. EM tomography is based on the tilting of sections in the electron beam and the mathematical analysis of collected data from many such tilt positions. The benefit of EM tomography is its ability to provide a high-resolution of the structures (5–10 nm) in three dimensions as the plastic sections are cut enough (200–1000 nm) to contain the sufficient amount of the information in the depth dimension. This is the most apparent difference comparing to the serial sections where the resolution in the depth dimension cannot exceed twice the section thickness (McEwen and Marko, 2001). The thickness of serial sections prepared by common procedures is around 70 nm and although Mastronarde et al. (1997) showed that serial sections can be cut as thin as 10 nm, the resolution is still 20 nm as opposed to 5–10 nm for EM tomography.

The expected results allowing distinguishing between the two different models of “sister” replisome organization are summarized in Fig. 1. The most relevant difference between the two models is represented by the change in the number of labeled domains after various length of the chase: while independent replisomes produce labeled domains the number of which is doubled at the latest during mitosis, the number of domains produced by the couples of replisomes is nearly quadrupled.

## 2 Materials and methods

### 2.1 Cell culture and synchronization

A human HeLa cell line was incubated in culture flasks or on coverslips in Dulbeco’s modified Eagle’s medium with l-glutamine (DMEM, Gibco) supplemented with 10% fetal calf serum (PAA Laboratories), 1% gentamicin and 0.85 g/l NaHCO_3_ at 37 °C in a humidified atmosphere containing 5% CO_2_.

For cell synchronization at the G1/S border, we used a double block with 2′-deoxythymidine (dT, Sigma–Aldrich Co., see Koberna et al., 2005). The cells were labeled with biotin-dUTP (Roche Diagnostics GmbH) or 5-bromo-5′-deoxyuridine (BrdU, Sigma Chemicals Co.) 100 min after they were released from the dT block. Further prolongation of the time after the release from the dT block showed that the replication pattern basically followed the schedule described earlier (Koberna et al., 2005), with some subtle differences in the timing observed, including a lower number of labeled foci in the 100-min experiments. During the several following tens of minutes, however, this number increased, approaching the one in the above-mentioned study. Nine hours later, the cells exited the S phase as more than 95% of them did not exhibit the BrdU signal. In some experiments, DNA synthesis was inhibited by means of aphidicolin. Cells were treated with 2 μg/ml aphidicolin (Sigma Chemicals Co.) for 2 h.

For the analysis of mitotic chromosomes, the cells were first synchronized by a double dT block, grown in a fresh medium for 100 min and labeled with biotin-dUTP (see below). After subsequent 9-h incubation in fresh DMEM, the cells were cultured for 5 h in a medium supplemented with 0.04 μg/ml nocodazole (Sigma–Aldrich Co., Zieve et al., 1980; in the presence of this drug, sister chromatids were separated with the exception of the centromere regions, Rieder and Cole, 1999). After these 5 h, most of the cells reached the mitotic phase as inferred from the shape of the mitotic cells and DAPI staining.

### 2.2 Labeling of the newly synthesized DNA and light microscopy detection of the labeled DNA

BrdU or biotin-dUTP were used as the markers of the newly synthesized DNA. In the case of BrdU, the cells were incubated in DMEM supplemented with 20 μM BrdU for 10 min and processed for LM (Masata et al., 2005).

The hypotonic approach was used to deliver biotin-dUTP into the cells (Koberna et al., 1999). This method makes it possible to treat a high number of cells and according to results published earlier does not cause physiological changes resulting in alternations of the replication dynamics (Koberna et al., 2005). In short, the cells were quickly rinsed with a hypotonic buffer (30 mM KCl, 10 mM Hepes, pH 7.4) and incubated in this buffer containing 0.2 mM biotin-dUTP alternatively for 4, 5, 10 or 15 min. The cells were subsequently fixed or incubated in a normal medium for 10 min unless otherwise stated, fixed and processed for LM or EM (Koberna et al., 1999). On the basis of the results of the LM analysis of the replication signal, we found that no incorporation occurred during the hypotonic treatment of biotin-dUTP and that the replication signal grew from 4- to 10-min incubation, in contrast, 10- and 15-min introduction of biotin-dUTP resulted into similar signal (not shown). Therefore, a 10-min delivery step was used in all experiments. Although we tried to increase the pool of biotin-dUTP and prolong the time of biotin-dUTP incorporation by increase of the concentration of biotin-dUTP in hypotonic buffer, we did not received standard results. Instead, the high variability both between individual cells and experiments was observed. In the next series of experiments, we incubated cells with introduced biotin-dUTP for 5, 10, 15 and 30 min in the medium. The cells were further processed for LM (Koberna et al., 1999). The signal increased between 5 and 10 min and stayed the same from 10 to 30 min incubation (not shown). On the basis of these results, it is apparent that the 10-min hypotonic incubation with biotin-dUTP under the applied conditions produced a pool of biotin-dUTP depleted during the first 10-min incubation in the medium. It enabled us to use biotin-dUTP in pulse-chase experiments with various length of chase.

The cells were viewed using a confocal microscope Zeiss LSM 5 DUO (Carl Zeiss Inc.) running on the LSM software 4.2. Plan Apochromat objective 100× 1.4 NA was used for the image acquisition. Fluorescence signals of Cy3 (excited at 561 nm using a Solid State Diode Laser) were detected using 575–615 nm emission filters. In all the experiments, we disregarded multinuclear cells as well as cells with large nuclei apparently possessing highly elevated genome copies, which were occasionally seen in the culture.

### 2.3 Antibodies

Mouse anti-bromodeoxyuridine antibody (Roche Diagnostics GmbH) and rabbit anti-biotin antibody (Enzo Biochem Inc.) were employed as primary antibodies. For LM, secondary antibodies conjugated with Cy3 (Jackson Immunoresearch) were utilized. For EM, we used the secondary antibodies conjugated with ultra-small (1 nm) gold (Aurion).

### 2.4 Electron microscopy and the evaluation of tomograms

All EM localizations employed synchronized cells. The ultrastructural mapping of the newly synthesized biotin-labeled DNA was achieved by means of the pre-embedding approach as described in Koberna et al. (2005). This method yields 3D information about the organization of the labeled DNA segments as the antibody labeling of replicated segments is performed before the embedding of cells into resin and therefore, the signal is inside the section not only on its surface as in the case of post-embedding labeling (Koberna et al., 2005). It is important that this approach does not result in noticeable changes in the organization of the tagged segments, as confirmed by post-embedding localizations (Koberna et al., 2005). Ultra-thin sections (70 and 200 nm thick) were cut on a Leica UltraCut S microtome (Leica Microsystems) with a diamond knife (Diatome Ltd.). The sections were stained with 3% uranyl acetate and viewed with electron transmission microscope Morgagni 268 (FEI Company) equipped with Megaview II camera (resolution 1280 × 1024 pixels) and Tecnai G2 Sphera tomography microscope (FEI company) equipped with Gatan Ultrascan 894 US1000 camera (resolution 2048 × 2048 pixels).

The 70 nm thick serial sections were cut as a ribbon of three or more adjacent sections and viewed in Morgagni microscope. Their position was mutually adjusted using the Adobe Photoshop software. The pictures of 70 nm thick sections were taken at magnification 14,000×.

EM tomography, from 200 nm sections, was performed at 200 kV by taking a tilt series of angular projections from −64° to +64° with an increment of 2°. The pictures were taken using a Gatan Digital Micrograph with the FEI Automated Tomography software at magnification 5000×. All the images were corrected for gain bias and dark noise. Such picture series were reconstructed using the IMOD software package (Kremer et al., 1996). The final 3D models were created using Amira software. To achieve more precise measurements, each side of the original tomogram had 10–20 nm cut off so as to minimize possible inaccuracies on the tomogram edges. When analyzing the size of the labeled domains, 300–500 labeled domains were measured in a 3D model while excluding the domains traversing the model faces. Although in principal our data can tend to underestimate the domains size it does not seem to be that case as the large fraction of labeled domains was completely trapped in the section volume and screened during 3D tomography analysis. This conclusion was also confirmed by our data from the analysis of serial sections as the majority of domains found in the middle section disappeared in one or both adjacent sections (not shown). The length of the labeled domains was measured as the longest distance between the outer edges of the silver deposits. When analyzing the number of labeled domains, on the other hand, the domains traversing the left, bottom and front faces of the model section were not considered. 100 sections of more than 50 different cells were analyzed in each experiment.

The total volumes of the cell nuclei in the early S-phase cell and mitotic-cell volume were calculated by means of Cavalieri’s estimator (Gundersen et al., 1988). The volumes were determined from serial sections by summing up the nuclear or cellular areas of all sections and multiplying the result by the section’s thickness. Fifteen cells from three different experiments were evaluated.

To evaluate the distance between doublets of labeled domains we identified as doublets only pairs of labeled domains with the similar size (the difference of the length was less than 20%), the similar labeling intensity (the difference of the labeling was less than 25%) and the similar shape. The statistical distribution of the measured distances between the doublets of the labeled domains from the 2-h experiment was fitted to the logarithmic normal peak described by the equation: $y=y_{0}+a*e^{\left[-0.5{\left(\frac{\ln\left(\frac{x}{x_{0}}\right)}{b}\right)}^{2}\right]}$, where *y* is equal to the frequency of the distribution of the individual distances and *x* to the distances between the doublets. Parameter *a* represents the high of the peak, parameter *b* the width of the peak, *x*_0_ corresponds to the peak maximum and parameter *y*_0_ to the additive constant. The amount of domains forming doublets was determined as a percentage share of the domains in pairs to the overall number of domains for each of the pulse-chase experiment. In that case only pairs of domains with the above-mentioned criteria and the mutual distance less than 400 nm were considered as doublets. The distance 400 nm represents approximately the upper border of the interval with the increased frequency of doublets deduced from the graph in Fig. 3.

## 3 Results

### 3.1 Approximately 5400 domains per cell nucleus were labeled after a 10-min labeling pulse

First, we determined the size and number of the labeled domains containing tagged segments in HeLa cells immediately after a 10-min pulse of biotin-dUTP, building on our previous study that showed that tagged segments are organized into small domains, which can be observed throughout the S phase (Koberna et al., 2005). In the early S phase, the labeled domains were randomly scattered in the cell nuclei except for nucleoli, and their distribution exhibited a tendency to aggregate progressively in the later stages of the S phase (Koberna et al., 2005). Such aggregation would complicate the resolution of the individual domains; therefore, exclusively cells synchronized into the early S phase were employed in this study.

A 3D reconstruction of the early S-phase cells showed that more than 90% of the overall signal in the form of silver-enhanced gold particles was clustered. On average, such clusters contained 7 ± 3 particles. A low scattered signal was observed in identically processed non-replicating control cells (not shown). Importantly, the labeling pattern reminded the pattern of individual, non-clustered silver particles seen in S-phase cells. Only about 5% of observed silver particles formed pairs. The number of silver particles arranged in clusters consisting of three or more silver particles was less than 1%. Similarly, such a low labeling was obtained after the aphidicolin inhibition of the activity of DNA polymerase α (not shown). These control experiments indicated that the DNA replication, and not the repair S-phase specific replication, occurs in the most of the labeled domains in S-phase cells. On the basis of these results we identified only the clusters comprising three and more silver particles as DNA replication-labeled domains.

The average maximum size of the labeled domains was 113 ± 40 nm. As a consequence of the labeling protocol, the size of the tagged chromatin segments in such domains is smaller. To obtain a more precise estimation of the intrinsic size of the tagged segments, it is necessary to correct the size of the antibody complex and the silver-enhanced gold particle (see Table 1). After the correction, the maximum diameter of the chromatin-tagged segments in the labeled domain is then larger than 74 ± 45 nm. This value is consistent with the thickness of one or two pairs of 30 nm chromatin fibers held together by “sister” replisomes and cohesin molecules (cf. Fig. 1A1 and A2).

**Fig. 2:**
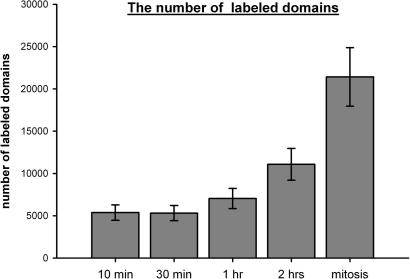
The number of the labeled domains. The average number of labeled domains per cell nucleus in 10- and 30-min, 1- and 2-h and mitotic experiments is shown. A similar number of labeled domains was found in the 10- and 30-min experiments. Approximately double and quadruple the number of labeled domains was found in the 2-h experiment and in mitotic cells, respectively. The data are represented as mean ± standard deviation.

On the basis of our data, approximately 21 labeled domains are present in 1 μm^3^ of the cell nucleus in the early S-phase cells after a 10-min pulse. As the total nuclear volume of an early S-phase cell calculated by means of Cavalieri’s method (Gundersen et al., 1988) was 260 ± 44 μm^3^, 5460 ± 923 domains were labeled in one cell nucleus after a 10-min incorporation of biotin-dUTP (Fig. 2). In fact, the number of the labeled domains of concurrently active replicons (the number of domains with an incorporation time approaching 0) is lower, because some of the domains contain tagged segments of replicons which began DNA synthesis during the pulse. The number of replicons that begin DNA synthesis during the pulse is inversely proportional to the replication lifetime of an average replicon and directly proportional to the labeling time. The lifetime of an average replicon can be estimated from the replication lifetime of a replication focus, because it represents a cluster of simultaneously active replicons (Zink, 2006 and citations therein). The lifetime of a replication focus is supposed to be approximately 1 h (e.g., Nakamura et al., 1986; Manders et al., 1992), which reflects the lifetime of the longest replicon in the cluster. Consequently, the average replicon lifetime should be shorter than 1 h. On the other hand, some of the tagged replicon segments beginning their synthesis in the last part of the 10-min pulse were not included in our data set due to the low signal of labeling. The first labeled domains can be observed shortly after the 2-min labeling pulse but are scarce and their size is smaller than the size of the domains after the 10-min pulse. Already a 3-min labeling pulse provides numerous domains with a size similar to the domains labeled for 10 min (Koberna et al., 2005). Supposing that the replication time of an average replicon is 1 h and the labeling time is 7 min (10 − 3 min), the estimation of the number (*n*) of the labeled domains of the currently active replicons (domains with a labeling time approaching 0) can be calculated according to the following equation: $n+\frac{7}{60}n=5460\pm 923$. Therefore, the number of the labeled domains after the correction is 4890 ± 827 and around 570 domains is initiated during the pulse. This calculation takes random gradual activation of the replication origins into account (Masata et al., 2005) although at this stage of the S phase the increase of the newly activated replicons may be faster. Although this value represents the rough estimation of the number of domains initiated during the pulse, it shows that this number represent only small portion (about 10%) of the overall number of domains containing active replicons.

Our data indicate that the used approach enabled us to distinguish the individual replicons as the number of labeled domains was several times higher as compared to the number of replication foci/factories published in previous studies (120–1500; Nakamura et al., 1986; Nakayasu and Berezney, 1989; Mills et al., 1989; Tomilin et al., 1995; Jackson, 1995; Jackson and Pombo, 1998; Ma et al., 1998). However, due to the lack of morphological differences between replication foci/factories and nucleoplasm, we were not able to directly address the organization of labeled domains in individual replication factories.

### 3.2 The number of labeled domains doubles after 2 h and quadruples after the complete sister chromatid separation in mitosis

**Fig. 3:**
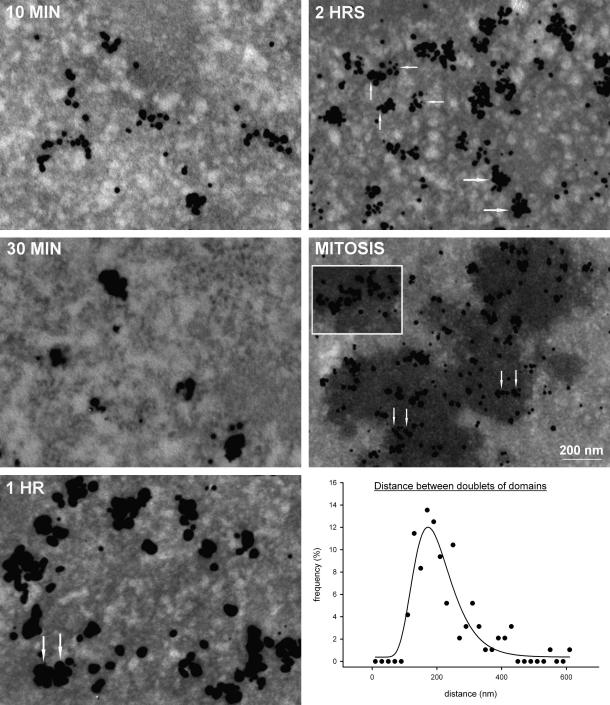
EM images of thin sections of HeLa cell nuclei with labeled domains and a graph of the distances between the doublets of labeled domains. Images of 70 nm-thick sections of the nuclei from the 10-min, 30-min, 1-h, 2-h and mitotic experiments are shown. The clusters of the silver-enhanced gold particles correspond to the labeled domains. The number of the labeled domains increases substantially between 1-h to mitotic experiments. The arrows in the images from the 1- and 2-h and mitotic experiments indicate the doublets of the labeled domains. The insert in the image of the mitotic-cell nucleus shows an example of a cluster of several labeled domains from a different cell. Seventy nanometers sections were chosen instead of 200 nm as they have higher contrast and accommodate much less number of labeled domains. In this respect, they are much more suitable for the demonstration of individual doublets although they cannot reflect their overall organization. Scale bar: 200 nm. The graph shows the frequency of the distances between the doublets of “sister” domains from the 2-h experiment.

**Fig. 4:**
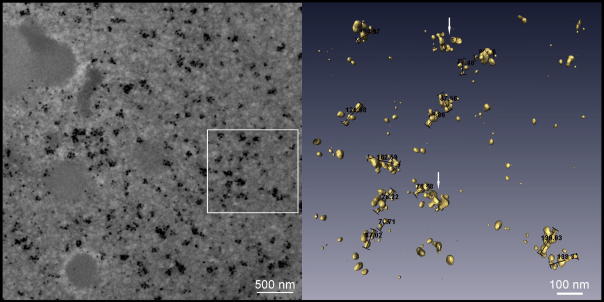
A 3D reconstruction of the labeled domains. The original image of a 200 nm-thick section of the cell nucleus from the 2-h experiment is shown on the left (Scale bar: 500 nm), whereas a 3D reconstruction of the labeled domains reconstructed from the insert is shown on the right (Scale bar: 100 nm). Only clusters of silver-enhanced gold particles in the outlined area of the electron microscopy image were reconstructed using Amira software. The length measurement is demonstrated. The arrows indicate labeled domains traversing the section faces.

Subsequently, we determined the number of labeled domains in the pulse-chase experiments. Biotin-dUTP was introduced into synchronized HeLa cells and the cells were incubated in the medium for 30 min, 1 h, 2 h and until mitosis (Fig. 2 and EM images in Fig. 3; an example of a 3D-reconstructed part of a 200 nm section is shown in Fig. 4). Our results showed that no incorporation of biotin-dUTP occurred during the hypotonic delivery step. Moreover, biotin-dUTP was incorporated into DNA only during the first 10 min of the incubation in the medium (see Section 2). Consequently, the chase corresponded to the 20-min, 50-min and 110-min incubation in the case of 30-min, 1-h and 2-h incubation in medium, respectively. Concerning of the mitotic cells, the chase was approximately 14 h (see Section 2). For the sake of simplicity, we will further indicate these pulse-chase experiments as 10-, 30-min, 1-, 2-h and mitotic experiment. EM tomography analysis showed that during the 30-min experiment, the number of labeled domains did not increase when compared with the 10-min experiment (Fig. 2; EM images in Fig. 3). This result clearly confirmed that biotin-dUTP is depleted from the cell’s pool during a time interval shorter than or equal to 10 min, since the 30-min experiment did not result in an increase in the number of domains as a result of the prolonged incorporation of biotin-dUTP into the replicated DNA of newly initiated replicons. On the other hand, we found a gradual increase in the number of domains in 1- and 2-h experiments. Around 7040 ± 1191 and 11,000 ± 1875 domains per cell nucleus were labeled in 1-h and 2-h experiments, respectively (Fig. 2, EM images in Fig. 3).

A detailed analysis of the replication pattern in tomograms from the 1-h experiment and especially the 2-h experiment revealed the occurrence of the domain doublets of a similar labeling intensity and shape (EM images in Fig. 3). We measured the distance between the centers of the domains in doublets in 2-h experiment (graph in Fig. 3). The 2-h experiment was chosen as the cells from this experiment clearly exhibited the highest incidence of doublets. Only pairs of labeled domains with the similar size, the similar labeling intensity, the similar shape and with the mutual distance under 1 μm were identified as doublets and evaluated. The average distance was approximately 227 ± 96 nm. If the lifetime of an average replicon is assumed to be around 1 h (e.g., Nakamura et al., 1986; Manders et al., 1992) and the speed of the replication fork movement around 0.6 kb/min in the early S-phase cells (Malinsky et al., 2001), the average size of a replicon in this phase should not exceed 72 kbp. As the length of a 2.6 kb-long fragment of completely stretched DNA is around 1 μm (Jackson and Pombo, 1998) and the packing ratio of the 30 nm fiber is ca. 40 (Wagner et al., 2005), the length of 72 kbp replicons in the form of 30 nm fiber corresponds approximately to 700 nm. The measured distance therefore corresponds to roughly one third of such replicon in the form of 30 nm fiber. If we suppose that each domain of this doublet represents a pair of tagged segments belonging to the same replicon, this is a very realistic value, indicating that at the level of replicon, the 30 nm fiber can be the highest level of chromatin condensation at least shortly after its replication.

Interestingly, we did not observe substantial differences in the intensity of labeling between the 10-min experiment and the 2-h experiment although the number of segments per domain was progressively reduced in the 2-h experiment when compared with the 10-min experiment. This may reflect the lower accessibility of biotin-dU epitope to antibodies in the newly replicated chromatin in comparison with the later stages. It can result from the presence of the large number of replication proteins masking the biotin-dU.

What is more important that the above-mentioned increase of the number of labeled domains favor the model of replisome couples (Model A2 in Fig. 1). The extremely large increase in the number of labeled domains in the 2-h experiment when compared with the 10-min experiment would suppose a complete separation of sister chromatids in the case of the model of replisome singles. Around 10%, i.e., 570 out of the 5460, labeled domains began DNA synthesis in the presence of biotin-dUTP (see above). Based on the model of replisome couples, the tagged segments of these replicons cannot contribute to the increase in the number of labeled domains by separating the segments originally bound by a replisome complex, because the segments initiated during the 10-min pulse are not separated by a non-labeled DNA strand. Consequently, the expected number of the labeled domains containing pairs of tagged segments of sister chromatids after complete separation corresponds to a doubling of 4890 (the difference between 5460 and 570) plus 570. Thus, the expected number is 10,350. This value is corroborated by the roughly 11,000 labeled domains found in the 2-h experiment. Moreover, for this model the higher number of labeled domains in the 2-h experiment can be explained by a partial separation of the sister chromatids.

To decide whether the model of tightly associated replisomes was correct, we counted the number of domains in metaphase cells after almost complete segregation of sister chromatids (Rieder and Cole, 1999).

**Fig. 5:**
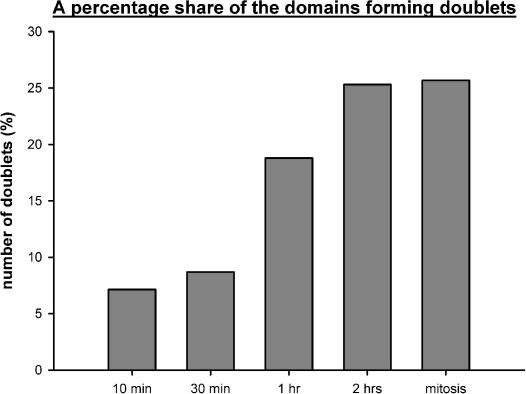
A percentage share of the domains forming doublets. A percentage share of the labeled domains forming doublets with respect to the overall number of domains is shown in the graph. The number of labeled domains increases between 30-min and 1-h experiments and between 1-h and 2-h experiments.

Approximately 11 labeled domains are present in 1 μm^3^ of the mitotic-cell volume. Since the mitotic-cell volume calculated by means of Cavalieri’s method (Gundersen et al., 1988) is ∼1919 ± 310 μm^3^, the total number of domains in mitotic cells was considered to be equal to 21,109 ± 3420 (Fig. 2, EM images in Fig. 3). The expected maximum number of labeled domains for the model of replisome couples based on the above-mentioned estimation of replicons labeled during a labeling pulse is 20,700 (4 × 4890 + 2 × 570) after the sister chromatids have segregated in mitosis. It is in good agreement with the measured value. The validity of the model of couples of “sister” replisomes was also supported by the observation of the doublets of labeled domains similar to those found in the 1-h and 2-h experiments. However, mitotic doublets were less apparent than doublets in the cells from the 2-h experiment as their identification was obscured by the occurrence of clusters of tightly associated labeled domains (EM pictures in Fig. 3). In order to compare the incidence of doublets in different pulse-chase experiments, we analyzed the number of labeled domains forming pairs in 10-, 30-min, 1-, 2-h and mitotic experiments (Fig. 5). Only pairs of labeled domains with the similar size, the similar labeling intensity, the similar shape and with the mutual distance under 400 nm were identified as doublets. The distance 400 nm represents approximately the upper border of the interval with the increased frequency of doublets deduced from the graph in Fig. 3. The analysis showed that the number of labeled domains forming doublets was the same in 10- and 30-min experiment, increased substantially between 30-min and 2-h experiments and remained similar in mitotic experiment (Fig. 5). This result is in complete agreement with the model of tightly associated replisomes. The measured number of paired domains in 10- and 30-min experiment probably represents the “threshold” level corresponding to the accidental occurrence of the pairs of similar domains.

### 3.3 The labeled domains maintain a similar size for at least 2 h after replication

**Fig. 6:**
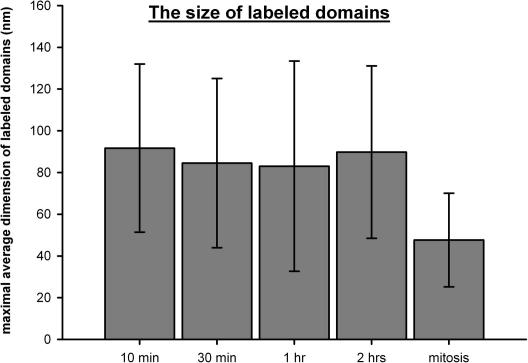
The size of the labeled domains. The maximum average size of the labeled domains from the 10- and 30-min, 1- and 2-h and mitotic experiments is shown. The labeled domains exhibit nearly the same maximum size in the 10-min to 2-h experiments. The maximum size of the domains in the mitotic cells was approximately two times smaller. The data are represented as mean ± standard deviation.

The size of the labeled domains was analyzed in order to describe the differences in the organization of the tagged segments in further detail. The average maximum size of the labeled domains was 113 ± 40 nm, 111 ± 40 nm, 120 ± 50 nm, 112 ± 41 nm and 66 ± 22 nm for the 10-min, 30-min, 1-h, 2-h and mitotic experiments, respectively. The size of the silver particle was subtracted from the diameter of the labeled domain measured for each of the experiments, because the size of silver-enhanced gold particles in the above-mentioned experiments differed. The corrected size of the labeled domains was 92 ± 45 nm, 85 ± 46 nm, 83 ± 58 nm, 90 ± 48 nm and 48 ± 26 nm (Fig. 6, Table 1). The similar size of the domains in the 10-min to 2-h experiments is in agreement with the suggestion that the maximum size of the labeled domains corresponds to the thickness of one to two pairs of tightly associated 30 nm fibers. This suggestion is also corroborated by the insensitivity of the maximum size of the labeled domains to the incorporation time of biotin-dUTP in the case of a 3- and 10-min labeling pulse (Koberna et al., 2005). According to the model of replisome couples (Fig. 1A2), two pairs of 30 nm fibers can be found in most labeled domains in the 10-min experiment. Later, as the segment pairs are moved away from the replisomes and the loop is finally relaxed, each labeled domain contains only one pair of the segments. In mitotic cells, only one labeled segment is accommodated in the labeled domain. The reduction in the number of segments in the individual domains between the 2-h and mitotic experiments is reflected in the steep decrease in the size of the domains labeled. Such a decrease was not observed between the 10-min and 2-h experiments, likely due to the similar thickness of the bundle of 4 or 2 parallel segments.

### 3.4 Zipping couples

**Fig. 7:**
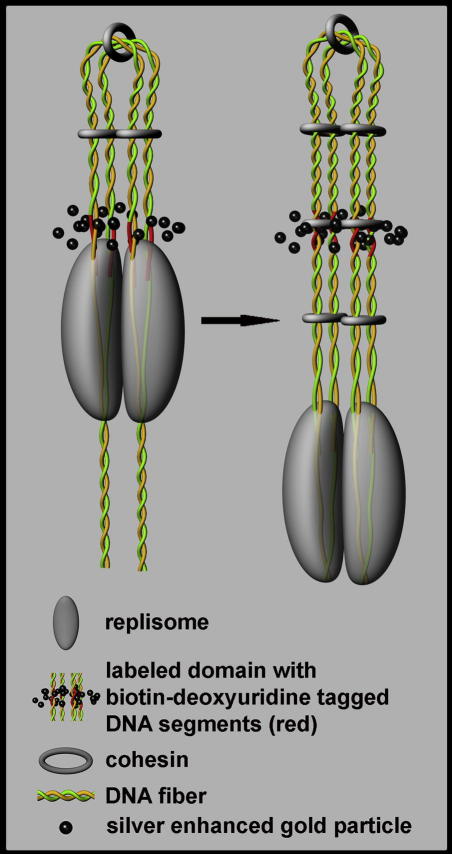
The model of zipping loops. Zipping of a DNA loop is shown. During replication, replisome couples produce a loop with the associated (zipped) arms probably in the form of four tightly associated 30 nm fibers. According to this model, “sister” pairs of biotin-dU-tagged segments of chromatids do not separate before the termination of the DNA synthesis of the replicon and the relaxation of the zipped arms. Immediately after labeling, the four tagged segments are present in one labeled domain (the left part of the image). Such an organization of the tagged segments persists during the synthesis of the whole replicon (the right part of the image). Although the mutual changes of the replisome position between left and right part of the Figure can result in the impression of movement of replisome along DNA, this model does not reflect whether DNA or replisome complex are moving during replication.

The more than doubled number of labeled domains in the 2-h experiment when compared to the 10-min experiment, the additional approximately twofold increase in the number of the labeled domains in mitotic experiment, the similar size of the domains in the 10-min to 2-h experiments and the substantial reduction in the size of the labeled domains in mitotic experiment strongly support the model of replisome operating as couples (Model A2 in Fig. 1). Another important finding concerns the arrangement of the DNA loop produced during the replication (see Fig 1): no increase in the number of the labeled domains in the 30-min experiment in comparison with the 10-min experiment suggests that the arms of the replicon loops are zipped to one another during replication and also for a certain time after termination of replication. According to these data, we propose a model of the arrangement of the newly replicated DNA into a bundle of four tightly associated 30 nm fibers (Fig. 7). Even though changes in the organization of chromatin as a consequence of our treatment of the samples are expected, the fact that neither the number nor the size of the labeled domains was substantially altered in the 30-min experiment with respect to the 10-min experiment argues against a complete collapse of the loop structure. The possible collapse would likely result in a higher number of domains and/or a different domain size, because only a specific parallel orientation guarantees that the labeled domains would have the same widths.

## 4 Discussion

We have used EM tomography to test two models of the organization of “sister” replisomes in human HeLa cells. Unlike the method used in the well-designed study concerning the organization of replisomes in yeast cells published earlier (Kitamura et al., 2006), the approach used in the present study made it possible to analyze the wide population of replicons in cells, which implied a high statistical relevance of our conclusions. Moreover, as compared with approaches requiring changes in genome information and manipulation of the protein environment, including the presence of fluorescent proteins, the applied EM approach resulted in a less extensive in vivo interference with the native chromatin organization as no changes in the protein composition of the tracked segments in living cells were necessary for their visualization. In this respect, the EM tomography analysis of the pre-embedding localization of the tagged DNA segments turned out to be a convenient tool for the high-resolution analysis of replicon organization in situ.

Our findings indicate that “sister” replisomes are arranged in tightly associated couples. Our results concern only human HeLa cells, but considering that the findings on prokaryotic cells (Lemon and Grossman, 2000; Jensen et al., 2001; Lau et al., 2003; Migocki et al., 2004) and budding yeast *S. cerevisiae* (Kitamura et al., 2006) were similar, such an arrangement is likely to be a general feature of DNA replication. Moreover, we present a modified model for replicon synthesis. According to our results, arms of a DNA loop formed during replicon synthesis are zipped to one another. The reason for such zipping as well as its mechanism remains unclear. Furthermore, it is not clear whether this phenomenon is also shared by other eukaryotic organisms. The rearrangement of such zipped DNA loops started at first 30 min after termination of replicon synthesis and is finished about 2 h after replication has finished. Our data also showed that the labeled DNA of sister chromatids is tightly held together by cohesin complexes at least for approximately 2 h after labeling. This is consistent with the findings about the cohesion of sister chromatids as the cohesion is apparently established in the S phase, maintained until mitosis when it is disrupted in the two step process (e.g., Uhlmann et al., 1999; Ciosk et al., 1998; Nasmyth et al., 2000; Hauf et al., 2001; Nakajima et al., 2007).

Although our data enable us to propose the model of replisome couples in human HeLa cells, they do not allow solving another important question: whether DNA or replisomes are moving during replication. This issue still remains a challenging point to be addressed although results of Kitamura et al. (2006) suggests that in yeasts DNA is moving rather than replisomes, and Hozak et al. (1993) showed results indicating that DNA is extruded from replication factories in HeLa cells.

In contrast, our data allow us to speculate on the arrangement of interphase chromatin. While several models of interphase chromatin organization have been suggested to date, a generally accepted model is still missing. This is caused by the difficulties associated with a high-resolution visualization of DNA organization in preserved cell nuclei, the necessity to obtain a 3D data set and enormous dynamics and plasticity of chromatin (Kurakin, 2007; Misteli, 2007). Basically, three models of interphase chromatin arrangements above the level of a 30 nm fiber have been suggested: a chromonema fiber model (Belmont and Bruce, 1994; Belmont et al., 1999), a giant-loop model (Yokota et al., 1995; Münkel et al., 1999) and a condensed-radial-loop model (Paulson and Laemmli, 1977; Marsden and Laemmli, 1979; Adolph, 1980). Belmont and Bruce (1994) observed chromatin fibers in the early G1 phase and the late G1/early S phase and denoted them chromonema fibers. In this model, a chromosome fiber of a diameter of ca. 100 nm folds into prophase chromatids of a diameter of 200–300 nm, which in turn coil into a metaphase chromosome structure (Belmont et al., 1999). The giant-loop model was suggested by a statistical analysis of the mean separation between two chromosomal sites as a function of genomic distance (Yokota et al., 1995; Münkel et al., 1999). The model of radial loops represents the extrapolation of the model suggested for mitotic chromosomes (Paulson and Laemmli, 1977; Marsden and Laemmli, 1979; Adolph, 1980). On the other hand, the model based on an electron-spectroscopic imaging of the nuclear chromatin provided a completely different view of the organization of interphase chromatin (Bazett-Jones and Hendzel, 1999; Dehghani et al., 2005). According to this model, the majority of the nuclear chromatin are composed of 10 nm and 30 nm chromatin fibers organized into lattices connecting inter- and intra-chromosomal space to form an almost contiguous nucleoplasmic space. Despite the fact that our results can reflect the chromatin organization only at the level of individual replicons, the distance between the frequently observed doublets of the labeled domains indicates that a significant portion of interphase chromatin is organized in the form of 30 nm chromatin fibers without additional packing at least shortly after replicon synthesis has been completed. This finding correlates with the above-described lattice model of chromatin (Dehghani et al., 2005) and also with the model of radial giant loops consisting of 30 nm fibers (Paulson and Laemmli, 1977; Marsden and Laemmli, 1979; Adolph, 1980). On the other hand, our results reflect only the organization of chromatin in the early S-phase cells and could not address the organization of chromatin replicated later in S phase.

Taken together, our data are in agreement with the model of replisomes operating as couples. Moreover, our data provide an indication of a specific organization of the replicated DNA in the form of a zipped loop of DNA, possibly in the form of 30 nm fiber.

## Figures and Tables

**Table 1:** Correction of the size of labeled domains for the antibody size and silver-enhanced gold particle.

Experiment	The measured size of labeled domains (nm)	The measured size of silver-enhanced gold particle (nm)	The size of labeled domains after correction of silver-enhanced gold particles (nm)	The size of labeled domains after correction of silver-enhanced gold particles and the antibody complex (nm)
10-min	113 ± 40	21 ± 5	92 ± 45	74 ± 45
30-min	111 ± 40	26 ± 6	85 ± 46	67 ± 46
1-h	120 ± 50	37 ± 8	83 ± 58	65 ± 58
2-h	112 ± 41	22 ± 7	90 ± 48	72 ± 48
Mitotic	66 ± 22	18 ± 4	48 ± 26	30 ± 26

The measured size of labeled domains was corrected for the size of silver-enhanced gold particle and the size of antibody complex. Only one size of the silver-enhanced gold particle was subtracted as the majority of its size represents silver deposits added to 1 nm gold particle already bound to the secondary antibody. The correction was performed according to the calculations deduced from analysis of domains observed in mitosis. The maximum size of the labeled domains is 66 ± 22 nm for mitotic cells and 48 ± 26 nm after reduction for the silver-enhanced gold particle. As there is no doubt about the existence of a 30 nm chromatin fiber in mitotic chromatin, the reduction should not be greater than 18 nm (48 − 30 nm) for the primary and secondary antibody.
